# Umbelliprenin, a bioactive constituent from the genus *Ferula* has cytotoxic and apoptotic activity in a dose- and time-dependent manner

**Published:** 2020

**Authors:** Seyed Ali Ziai, Omid Gholami

**Affiliations:** 1 *Department of Pharmacology, School of Medicine, Shahid Beheshti University of Medical Sciences, Tehran, Iran*; 2 *Cellular and Molecular Research Center, Faculty of Medicine, Sabzevar University of Medical Sciences, Sabzevar, Iran*

**Keywords:** Umbelliprenin, Apoptosis, Dose-response

**Dear editor**

We praise the article by Iranshahi et al. (2018) ▶, entitled “A review on the cytotoxic activity of the genus *Ferula* and its bioactive constituents” published by Avicenna Journal of Phytomedicine (Iranshahi et al., 2018 ▶). It was a well-designed and interesting review article on the cytotoxicity and apoptosis inducing activity of *Ferula* species and their phytochemicals in cancerous cell lines and their possible mechanisms of action. Here we want to add some notifications about umbelliprenin, one of the phytochemicals mentioned in the article. As it was mentioned in the article, umbelliprenin is a prenylated coumarin synthesized by various *Ferula* species like *F. szowitsiana*. Umbelliprenin has a structure close to that of auraptene, another prenylated coumarin from *Ferula* species. The only difference is the higher length of the 7-prenyloxy chain which contains 15 instead of 10 carbons (Barthomeuf et al., 2008 ▶) (Figure 1). 

Umbelliprenin has different pharmacological effects such as cytotoxic and apoptosis inducing activities (Ziai et al., 2012 ▶; Shakeri et al., 2014 ▶; Sattar and Iranshahi, 2017 ▶; Naderi Alizadeh et al., 2018 ▶; Rashidi et al., 2018 ▶). 

Although authors correctly mentioned that umbelliprenin induced the extrinsic and intrinsic pathways of apoptosis in the text, but in Figure 2, they mentioned that umbelliprenin only has mitochondrial (intrinsic) mechanism (Iranshahi et al., 2018 ▶). It should be noted that umbelliprenin induces both intrinsic and extrinsic pathways of apoptosis (Gholami et al., 2013 ▶).

**Figure 1:**
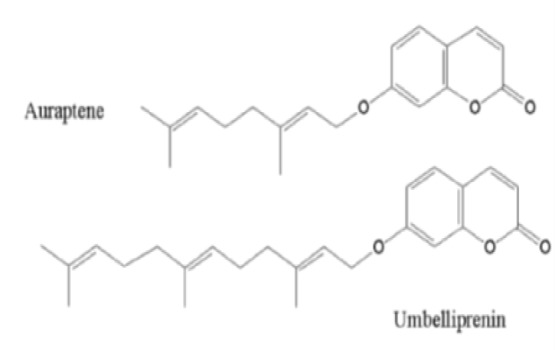
Chemical structure of auraptene and umbelliprenin

As it is mentioned in the article, we showed that umbelliprenin induced apoptosis in leukemic cell lines. dose- and time- dependently (Ziai et al., 2012 ▶). Interestingly, we noticed that umbelliprenin and some other phytochemicals show this dose-response relationship by hormesis phenomenon (Gholami, 2017 ▶; Kafi et al., 2018 ▶). 

The hormetic dose-response relationship becomes the object of considerable investigations on a broad range of chemicals over the past 2 decades (Calabrese, 2013 ▶). In this sense, a compound may have opposite effects at small vs. large doses. Study on hormesis phenomenon in induction/inhibition of apoptosis by natural compounds like umbelliprenin is still at the beginning of its path and it is the subject of our future studies.

In the end, we congratulate Iranshahi et al. for their article and we appreciate Avicenna Journal of Phytomedicine editorial board for their judicious concern on this topic. We are looking to read well-original and review articles regarding the beneficiary effects of the genus *Ferula* and its bioactive constituents in future.

## Conflict of interest

The authors declare no conflicts of interest. 
